# Adaptation and validation of the Spanish version of the DOS questionnaire for the detection of orthorexic nervosa behavior

**DOI:** 10.1371/journal.pone.0216583

**Published:** 2019-05-07

**Authors:** María Laura Parra-Fernández, María Dolores Onieva-Zafra, Juan José Fernández-Muñoz, Elia Fernández-Martínez

**Affiliations:** 1 Department of Nursing, Physiotherapy and Occupational Therapy, University of Castilla-La-Mancha, Ciudad Real, Spain; 2 Department of Psychology, Universidad Rey Juan Carlos, Alcorcón, Madrid, Spain; 3 Department of Nursing, University of Huelva, Spain; Universitat Hohenheim Fakultat fur Naturwissenschaften, GERMANY

## Abstract

**Introduction:**

Orthorexia nervosa, a term used to describe an obsession with healthy eating, has been shown to have major health implications for those affected. The aim of this study was to validate a Spanish version of the Düsseldorfer Ortorexie Skala (DOS), a questionnaire for the detection of orthorexic behavior.

**Methods:**

A cross-sectional study comprising a total sample of 492 Spanish participants recruited from the University of Castilla-La Mancha, Spain, and randomly divided into two groups. The following tools were applied: the DOS and the Eating Disorder Inventory (EDI-2). The factorial structures were analyzed using exploratory and confirmatory factorial analysis.

**Results:**

The internal consistency of the DOS-ES was α = .841. The exploratory factor analysis has revealed the existence of a single factor with factor loadings ranging from .508 to .802. A confirmatory factor analysis was applied to the second half of the random sample in order to confirm the factor solution.

**Conclusion:**

The Spanish adaptation of the DOS-ES is proven to be a reliable and valid questionnaire for evaluating the obsessive tendency towards healthy eating among university students.

## Introduction

Over recent years, there has been increasing research interest on the subject of orthorexia nervosa (ON), both regarding the definition and/or classification of this disorder, as well as the consequences and possible detection of the same [1,2]. This disorder is characterized by an obsession with healthy eating [3], and may cause severe physical, psychological and social impairment [4] in individuals who adopt this type of diet as a lifestyle, taking it, at times, to extremes that are considered pathological. During the 21^st^ century, as a consequence of the vast amount of information available, social networks sites are full of images of healthy food labels corresponding to various foods products or norms and/or diet guidelines which may not always be so healthy, and which may derive in behaviors at risk of developing ON [5,6].

At present, this potential disorder is not included in the principal manual of mental health disorders, i.e. the Diagnostic and Statistical Manual of Mental Disorders (DSM-5), and therefore, it is not classified as a mental health disorder. While there is a consensus among some authors to consider ON as an eating disorder (ED) [7–9], others have revealed many similarities between this disorder and Obsessive Compulsive Disorders (OCD) [10]. Additionally, some authors have found similarities between ON and autism spectrum disorders [8,11].

Little is known regarding the consequences of ON, considering that recent research has attempted to contextualize, define and relate symptoms or behaviors that are still largely unknown for this disorder. Moroze et al. described a possible clinical case of ON where this excessive concern for healthy eating was highlighted. This disorder may provoke severe consequences in individuals, including significant malnutrition, testosterone deficiencies, constipation, bradycardia, deficient dentition, osteoporosis, leucopenia, thrombocytopenia and metabolic alkalosis [4]. Recently, an article was published which cited two clinical cases in children where the clinical history of the parents displayed very similar characteristics to those found in individuals with ON. The study describes how the parents attempted to provide a healthy diet which only included vegetable based foods, without salt or condiments and excluding animal-based or packaged products [12]. However, the scarce amount of literature available refers to rather extreme cases, where the health consequences of this pathology are more obvious. Thus, the challenge is to be able to precisely identify the patients who are potentially at risk of developing ON, understanding that a milder or subclinical form of the same may hide important consequences in the long term.

Several tools have been developed to evaluate the presence of ON. The first to develop a measurement Tool was Bratman in 2001 who proposed The Bratman Orthorexia test (BOT), which was used to evaluate ON tendencies and eating attitudes [3]. However, this tool was shown to have limited use [13,14]. Later, in 2004, Donini et al. developed another diagnostic tool: the ORTO-15 for ON, which was based on the BOT [15] and used in Italy [16]. There have been multiple adaptations and validations of this questionnaire in other countries [17–21] This was a first step to provide researchers and professionals with ON scales for early detection purposes.

Currently, the most used tool is the ORTO-15 in its different versions. However, this is also the tool which has received the most criticisms on behalf of the scientific community. The reported epidemiology of ON, measured with this instrument, has been highly variable. The first study was performed by Donini et al. in Italy using a sample of 404 subjects who reported a prevalence of 6.8% [22]. If we compare this to one of the most recent studies performed by Dell’Osso et al. in Italy with a sample of n = 2130 students, 34.9% of subjects with a score below the cut-off point of 35 were found to be at risk of ON [23]. However, it is interesting how a study conducted in the United States in 2017 by Dunn et al. with a sample of 275 university students found that 71% of the sample scores were below 40, which is consistent with other studies. However, the authors pointed out that 80% of the sample had no food restrictions whatsoever, and moreover, the small subgroup identified as a vegans had an ORTO-15 score above the established cut-off point [2]. Many authors have reported that this variability in the data could be due to sociocultural aspects of the samples [18,24]. However, most have criticized the tool used in these studies [20]. Moreover, the ORTO-15 has also been criticized regarding its validity for measuring ON, as it includes elements that are not specifically characteristic of ON, shedding doubt on the apparent validity of the test [18]. Therefore, new tools are necessary to further study ON.

When epidemiological studies are performed, there is some considerable doubt concerning whether or not the measurement tools are able to provide valid and trustworthy measurements. In 2015, in Germany, Barthels et al. developed a new tool, the Dußeldorfer Orthorexie Scale (DOS) test, consisting of 10 items and with proven reliability, ranging from satisfactory to good, with an internal consistency of a = .84 [25]. At present, the first adaptation by Chard et al. is available in English. These results have been highly satisfactory with a Cronbach’s alpha of 0.88, thus demonstrating that this is a good tool for evaluating ON [26]. In the studies performed with this tool, the reported prevalence rates were approximately 9% of people at risk of developing ON in the study by Depa et al. [27] and 3% in the study by Strahler et al[28], thus considering ON as being a less common disorder, as suggested by other authors. It is important, to therefore avoid incorrectly labelling certain behaviors that are related with healthy eating habits as being ON[5,14].

The present study is based on the adaptation and validation of the Spanish version of DOS with the following aims: 1) to present a Spanish version of the DOS, 2) to determine the internal consistency of the scale, 3) to examine the unidimensional structure of the scale, and 4) to explore the relationship between DOS and other personality traits.

## Material and methods

The development of the preliminary version of the scale followed the usual recommendations [29]. After obtaining the permission of the original authors, two translations of the original version were made in the corresponding language of the target population on behalf of bilingual translators whose mother tongue was Spanish. A back-translation of the same was then performed by professionals whose mother tongue was German. To assess the comprehension and suitability of the questionnaire, a pilot study was performed with 20 Spanish-speaking participants who were students of the nursing faculty. Each participant was asked whether it was difficult to understand each item and whether it was easy to fully understand the questions. This resulted in the final version of the DOS-ES questionnaire.

### Sample

The questionnaire was subsequently applied to a convenience sample of 492 university students from the Ciudad Real campus of the University of Castilla–La Mancha, Spain, who were enrolled in the 2017–2018 academic year. The average age of the students was 19.97 years (SD = 3.03). Of these, 63.8% were students from Health Sciences while 36.2% were architecture and engineering students. The mean Body Mass Index was 22.64 (SD = 6.6).

The questionnaires were voluntarily completed using an electronic platform, and the anonymity of participants was ensured. The study was approved by the Ethics Committee of the University Hospital of Castilla-La Mancha, according to the ethical guidelines established by the Helsinki Declaration in 2008 (Code C- 153).

### Instruments

Demographic information was collected. Participants’ self-reported sociodemographic characteristics, including age, gender, weight and height.

The Eating Disorder Inventory (EDI-2) is a self-reported 91-item questionnaire, answered on a six-point Likert-Type-Scale using a three-point system where ‘sometimes’, ‘rarely’, and ‘never’, are assigned zeros while ‘often’, ‘usually’ and ‘always’ are assigned a score of 1, 2 and 3, respectively. The questionnaire is used to assess eating-disorder symptoms, attitudes and behaviors. It contains 11 subscales: drive for thinness, body satisfaction, bulimia, effectiveness, perfectionism, interpersonal disruption, interceptive awareness, maturity fears, asceticism, impulse regulation and social insecurity. The sub-scale scores can be calculated by simply adding the scores of all the items of each specific sub-scale. The EDI-2 total score ranges from 91 to 546. We used a Spanish version of the scale validated by Corral, Pereña and Seis-dedos in 1998 [30], which showed an internal consistency of 0.83–0.92.

The EDI-2 is widely used in Spain and has proven to be widely accepted as a valid instrument for the accurate diagnosis and detection of any eating disorder risk (EA) [31–33] among the Spanish population. We chose to use the EDI-2 based on its good psychometric proprieties in both clinical settings and non-clinical samples, [34] as well as the possibility it offers of separately assessing different dimensions [9,35].

The Düsseldorfer Orthorexie Skala (DOS) (Table 1)This is a scale to evaluate orthorexic eating behavior. This scale consists of ten affirmations of healthy eating behavior. The internal consistency with Cronbach Alpha is 0.83. This scale is based on a four-level scoring system from "this does not apply to me (1)" to "this applies to me” (4)'. The minimum score is 10, and the maximum score is 40 points. The preliminary cut-off point for orthorexia is suggested when there is an accumulated score of 30 points, or more, according to the 95th percentile of the design sample [26].

**Table 1:** DOS translation into Spanish.

	Spanish version
Item 1.-	Para mi, comer alimentos saludables es más importante que el placer
Item 2.-	He fijado reglas en mi alimentación
Item 3.-	Solo puedo disfrutar de la comida/los alimentos si estoy seguro de que son saludables
Item 4.-	Trato de evitar invitaciones a cenar a cas de amigos si no prestan atención a una alimentación saludable
Item 5.-	Me parece positive prestar más atención a una alimentación sana que otras personas
Item 6.-	Cuando he comido algo insano me hago grandes reproches
Item 7.-	Tengo la sensación de que soy marginado por amigos y colegas a causa de mis estrictas reglas de alimentación
Item 8.-	Mis pensamientos giran constantemente en torno a una alimentación saludable y adapto mi rutina diaria en consecuencia
Item 9.-	Me resulta difícil romper mis reglas en la alimentación
Item 10.-	Cuando he comido algo insano me siento hundido

### Statistical analysis

Statistical analyses were performed to calculate the Exploratory Factor Analysis (EFA) with SPSS 23.0 and the Confirmatory Factor Analysis (CFA) with AMOS 18.0 and FACTOR 10.8.03 (http://psico.fcep.urv.es/utilitats/factor/Download.html). Firstly, multivariate normality, linearity, high sample size and correlation between items were checked in order to apply these statistical procedures. Secondly, the estimation method was maximum likelihood because it is assumed that the distribution of the data was multivariate normal. A varimax rotation method was conducted and the KMO index and Barlett test of sphericity was calculated [36]. Finally, to identify the adequate number of factors, the Horn's parallel analysis [37] and the Cattel’s scree plot [38] were carried out in order to extract the optimal number of factors.

Considering the sample size, the same was randomly split into two subsamples. With the first subsample we ran an EFA. Once this revealed the most appropriate number of factors to represent the data, we conducted a CFA on the second randomly selected subsample in order to confirm that the factorial structure fitted the data. The overall fit of the factorial solution was confirmed with the absolute fit indices: chi-square statistic X2, and the Adjusted Goodness of Fit Index (AGFI) (where values over .90 imply an optimal model). In addition, the measures of absolute and relative fit used were: the Comparative Fit Index (CFI) and the Normed Fit Index (NFI) also named delta 1. In both cases, the range of values should be between 0 and 1, with scores over .90 involving a good model fit. Likewise, the Standardized Root Mean Square Residual (SRMR) and Root Mean Square Error of Approximation (RSMEA) were also checked on the overall fit. Thus, scores under .08 indicated a great overall fit of the model [39–41].

## Results

### Descriptive findings and internal consistency measurement

The internal consistency of the ES-DOS was α = .841 and the values of the total item correlation were significant and medium-to-large with a minimum value of .418 (item 9) and a maximum value of .670 (item 8). Moreover, all the bivariate correlations between the items were significant for p < .05 and p < .01 (.204< r < .625). Thus, Table 2 shows the descriptive analysis (mean, standard deviation, kurtosis and skewness) and the total item correlation for the ten items. Moreover, we have included the factorial weight for the EFA.

**Table 2:** Mean, standard deviation, item discrimination, kurtosis, skewness and reliability for the ten items of the Duesseldorf Orthorexia Scale (DOS-ES).

Item wording	M	SD	Kurtosis	Skewness	Item-total correlation	FW
Item 1.	2.21	.852	-.580	.251	.452	.531
Item 2.	2.33	.966	-1.01	.081	.573	.651
Item 3.	1.69	.836	.284	1.03	.582	.681
Item 4.	1.27	.588	5.41	2.33	.537	.668
Item 5.	2.36	.951	-.906	.136	.527	.606
Item 6.	1.79	.881	.044	.920	.612	.726
Item 7.	1.17	.461	9.65	2.97	.449	.583
Item 8.	1.61	.828	.645	1.21	.670	.770
Item 9.	1.62	.817	.530	1.16	.418	.522
Item 10.	1.46	.728	2.40	1.66	.627	.745

### Exploratory and confirmatory factor analysis

The Bartlett's test of sphericity was significant with a value of chi-square 841.663 (df = 45) p < .001 and the value of Kaiser-Meyer-Olkin (KMO) was .873. The exploratory Factor Analysis revealed the existence of a single factor with factor loadings ranging from .522 to .770 (Table 2). Likewise, the parallel analysis showed a unidimensional solution according to the set of items. The explained variance was 43.78% for the unique factor identified. The goodness of fit statistics for EFA were: AGFI = .96, CFI = .99, SRMR = .06 and Schwarz’s Bayesian Information Criterion (BIC) = 200.236. In order to test the factorial structure of the Exploratory Factor Analysis, a Confirmatory Factor Analysis was applied on the second half of the random sample to confirm the factor solution. The model achieved a good fit; all indices had scores near to the recommended values. Table 3 shows the results of the CFA corresponding to the second half of the sample: X^2^ = 64.93; X^2^/degree freedom = 2.095; AGFI = .912; NFI = .924; CFI = .958; SRMR = .028 and RMSEA = .067.

**Table 3:** Goodness-of-fit indices for Duesselforf Orthorexia Scale (ES-DOS) for the CFA.

χ2	p	χ2/DF	AGFI	CFI	NFI	SRMR	RMSEA
64.93	.000	2.095	.912	.958	.924	.028	.067

N = 244; AGFI: Adjusted Goodness of Fit Index; RMSEA: Root Mean Square Error of Approximation; CFI: Comparative Fit Index; SRMR: Standardized Root Mean Square Residual and NFI: Normed Fit Index.

### Criterion validity

The criterion validity of the DOS-ES scale was gathered via the analysis of the correlations between the subscales of the EDI. In this way, the correlations were significant for: drive for thinness (r = .500, p < .01), body dissatisfaction (r = .288, p < .01), bulimia (r = .195, p < .01), effectiveness (r = .160, p < .01), perfectionism (r = .176, p < .01), interoceptive awareness (r = .237, p < .01), asceticism (r = .331, p < .01), impulse regulation (r = .166, p < .01) and social insecurity (r = .092, p < .05). However, there was no significant correlation with the subscales: interpersonal disruption (r = .029, p > .05) and maturity fears (r = -.007, p > .05).

## Discussion

This study aimed to research the psychometric properties of the Spanish version of the DOS questionnaire among university students. We decided to validate this tool due to the lack of existing tools that measure a behavior comparable to ON in a Spanish population.

The DOS was satisfactorily adapted to the Spanish population using a rigorous procedure of translation and validation, which led to the Spanish version: DOS-ES.

The participants responded to all the elements via the use of online questionnaires. The responses that were scored for each item followed a normal distribution, therefore, no items were removed from the original DOS. A good global adjustment of the model was obtained, however maximum and minimum values were observed which exceeded the acceptable values. The minimum value was found on item 9, “It is difficult to break my diet”, which may be due to the non-specification or delimitation of the word “diet”. For example, this may be confusing due to the extent of its meaning and because it may be linked to other causes (digestive pathologies, intolerances, allergies…) which may not necessarily be related to healthy eating. This subject has already been discussed by the scientific community in other questionnaires developed to date for the detection of ON [20].

The results of the internal consistency of the tool are in consonance with the findings reported in the study of the original scale and also in line with a recent study published in the USA [25] [26]. Concerning the confirmatory factorial analysis performed in the present study, the DOS-ES questionnaire was found to present a unidimensional structure with a good fit for this one-factor model. In this sense, the results of our study are in consonance with one of the aims set out by the original creators of the questionnaire, which was to create a single-factor scale, which they were unable to justify. This is because the factorial validity for this unidimensional model could not be demonstrated. Therefore, this postulated unidimensionality was rejected, opening the possibility for new studies to reexamine the structure of the DOS questionnaire[25]. In the English validation of the questionnaire, the authors reported a poorly fitted one-factor model in contrast with a good fit for the five-factor model. However, while, statistically, a five-factor structure would have been preferred, the authors discarded this, understanding that this would ignore the clinical construct features of ON, plus they did not recommend a separation of items in the 10-item DOS [26]. Differences both in the methodology as well as in the population should be considered when establishing any hypothesis regarding this difference concerning the structure of the questionnaire.

Significant correlations were found between the total score of the DOS-ES and the different subscales of EDI-2 in nine of the eleven dimensions, in which drive for thinness obtained the highest correlation. In this sense, our study supports the relationship that exists between some of the discussed dimensions of the EDI-2 (drive for thinness, bulimia and body dissatisfaction) due to their possible point of convergence between ON and other EDs. One of the items which may suggest this close relationship within the dimension “drive for thinness” is item 1 *“I eat sweets and carbohydrates without worrying”*, where the marked difference between both disorders is based on the quality of the food for people at risk of ON and the amount (i.e. calories), for people with a diagnosis of AN (due to the relation of the same with weight gain). Indeed, the issue of the quality versus the quantity of food, has been highlighted by some authors as being the main difference between both disorders[4].

Among these, drive for thinness also shows the highest score in the correlation with the English validation study of the E-DOS in the study by Chard et al., with the aim of validating the same in English [26]. In a study performed in Germany with a sample of over 1000 patients diagnosed with some kind of mental disorder related to an affective, eating, anxiety or personality disorder, and using a different scale for the measurement of the risk of ON (the ORTHO-10), a positive correlation was also found with the score of the dimension drive for thinness, among others [42]. In a study performed with 32 patients with AN or BN, Segura et al. reached the conclusion that ON seems to be associated both with the clinical improvement of AN and BN, as well as the migration towards less severe forms of EDs [43]. The only clinical study using the DOS performed to date was conducted by Barthels et al. in patients with anorexia nervosa (AN), concluding that ON behavior may be a coping strategy in people with AN [9]. Kinzl et al.[44], suggested that ON could be the beginning of more serious EDs, such as AN. The main difference suggested by most of the literature consulted is that in patients with AN or bulimia nervosa (BN), their main concern is related to the consumption of the amount of food [45], and their obsession for controlling their body weight. This differs from individuals with ON who are more worried about the quality of the foods they eat without excessively worrying about the kilocalories of foods [46]. The main challenge for the scientific community is to clarify whether ON could have its own entity as an ED and become an independent disorder [3], or whether it can be categorized within the clinical evolution of other disorders [47].

The present study has several limitations which must be considered. First, both the DOS-ES as well as the EDI-2 are self-report tools and, as such, these may be less precise in the assessment of the symptoms of ON and EDs. This is because data that is obtained through self-report methods means its accuracy depends on the truthfulness of the respondents and their willingness to share experiences on this sensitive topic. This means that their responses may be prone to exaggeration or minimization according to the judgement of participants. Secondly, another limitation of the study is that 60 per cent of the participants studied Health Sciences. Perhaps, this population may be different in terms of eating behavior and interest in health and eating issues compared to students studying other subjects (e.g. other natural sciences such as mathematics or physics). Thus, in future studies, it would be necessary to examine the psychometric properties of the DOS-ES, as well as other parameters such as the divergent validity of the questionnaire in other population sectors in Spain, and to evaluate the usefulness of DOS-ES among other populations and also among clinical populations.

Despite these limitations, the present study provides preliminary validity and evidence of reliability for the Spanish version DOS-ES. In this sense, the DOS-ES offers a simple and economic tool which may facilitate far deeper research and larger epidemiological population- based studies which also enable transcultural comparisons.

## Conclusions

The Spanish adaptation of the DOS-ES proposed in this study shows a reliable and valid questionnaire for evaluating the obsessive tendency towards healthy eating among the adult population. Considering that Spanish is spoken by more than 572 million people worldwide, the Spanish version of the DOS-ES can be used effectively as an assessment in many contexts, involving a wide range of Spanish speaking populations. Further evidence should be gathered to explore its functioning in other contexts, such as clinical contexts.

## Supporting information

S1 File Supporting information. (XLSX)
